# Transactions of the Association of Army and Navy Surgeons

**Published:** 1864-06

**Authors:** 


### TRANSACTIONS OF ASSOCIATION OF ARMY AND NAVY SURGEONS.

Association of Army and Navy Surgeons, March 26, 1864.

Surgeon-General S. P. MOORE, President, called the meeting to order at 8 P. M.

The minutes were read and approved. A letter was read from Dr. J. A. Cunningham accepting the election as honorary member of the Association, and expressing his thanks for the honor conferred. Letters were also read from Surgeons Jno. A. Hunter, Medical Director, A. N. V., W. B. Harrell, P. G. Robinson, C. H. Ladd, W. M. Wilson, W. F. Richardson, and E. B. Haywood, on the subject of tetanus.

Surgeon CHAMBLISS exhibited a pathological specimen of interest, consisting of two aneurismal tumors, resulting from gun-shot injury to the deep-seated femoral artery.

The question before the association relating to idiopathic tetanus was read.

Surgeon MICHEL remarked that there appeared to have been no development of idiopathic tetanus from the atmospheric exposure through which our armies had passed. Notwithstanding the insufficiency of these influences in engendering this form of the disease, traumatic tetanus had prevailed to some extent. When originating from alternating degrees of heat, cold and moisture, he believed the disease was a *centric* disturbance. The traumatic variety was perhaps *eccentric*, at least, when some nerve sustained injury. Tetanus, after parturition, was possibly traumatic, for during this physiological act the uterus undergoes a complete exfoliation of its mucous surface. A vast surface entirely denuded of its investing membrane, presents a lesion readily productive of danger. The spontaneous development of tetanus is so rare that many denied the occurrence, so prone are we to impute this disease to some occult or ill-defined lesion of a nerve. There was no doubt, however, of its occurrence without any lesion. It had been ascribed to injury produced by worms, which he did not believe probable. Any of these forms or varieties he was disposed to regard as indicative of a blood disease. A dyscrasial condition of the blood was adequate to the production of such symptoms, and certainly accounted more satisfactorily for their vehemence and the fatal issue, even in traumatic cases, in which so seldom any appreciable lesion is discoverable. This view of the pathology of tetanus was not new; it is entertained by high authority, both Todd and Travers had advanced the doctrine that some poisoned state of the blood probably existed. One of these pathologists in search for the specific cause resorted to a test similar to that performed by Marshall Hall for the detection of strychnine. M. Hall having detected the thousandth part of a grain of strychnine, by immersing a frog in a prepared solution of the poison, rendering it thereby *tetanic*, or as he termed it, *strychnoscopic*. Dr. Travers endeavored to induce tetanoid symptoms in the same animal by subjecting it to prolonged contact with a concentrated solution prepared from the blood and effete products of the wound of a tetanic patient, without, however, any decisive result Though unsuccessful in this single experiment, it was worthy of repetition under the favorable opportunities now presenting themselves.

Surgeon SPENCE said he had known of the disease being brought on from simple exposure, and he would relate the case: In September, 1852, a servant was engaged in gathering fodder until late at night, when the patient fell accidentally asleep, being exposed all night in the open air. In this case no marks of injury of any kind could be discovered. The case recovered, after lasting sixteen or eighteen days. Resorted to cauterization of the whole spine, administering morphine, stimulants and nourishment. There was trismus well developed in this case.

Surgeon C. B. GIBSON observed that though he did not consider it of practical importance to discuss whether there was such a disease as idiopathic tetanus, yet he declared his disbelief in the existence of any such affection. Tetanus, he thought, was always of traumatic origin, the failure to discover the lesion in particular cases was no contradiction of its existence, for it might be concealed in situations not examined. An infinite series of causes might produce imperceptible injuries of nerves sufficient to induce an attack, since the simplest scratch is known to be sufficient. A case was on record of a servant who died in fifteen minutes from a small scratch, the result of the breaking of a dinner-plate. It was not improbable that some foreign body in the rectum, or even hardened fæces, might produce laceration of the mucous membrane during defecation. This would be enough to establish at once a sufficient cause. Some writers had denied the existence of idiopathic tetanus, and he was disposed to agree with them. Tetanus, after confinement, was obviously traumatic, not so much on account of the exfoliation of the uterine mucous membrane, which might be considered a physiological condition, as the accidental lacerations about the neck of the womb. Dr. G. said it was far more important to arrive at some conclusion as to the most efficient mode of treating this affection; he considered the remedies applicable to, and not the kind of tetanus as important. Patients died and recovered under all kinds of treatment. He would recommend some agreement, if possible, among the profession, as the most approved of methods of cure.

Surgeon SPENCE thought that the use of chloroform, both internally and externally, was always called for in combination with some stimulant. He had seen this plan prove serviceable, even in a horse. The animal had trismus, though no wound was discoverable, and by the active use of chloroform, two ounces of which were administered in one pint of whiskey, while inhalation was persevered in, the horse recovered.

Surgeon MICHEL said that after the discovery of anæsthetics, great hopes were entertained as to the successful treatment of tetanus by their use, but, unfortunately, experience had not realized these expectations. Where spasms were controlled, or even entirely ceased during their administration, patients died apparently from nervous exhaustion, which circumstance confirmed the belief in some blood cases. Anæsthesia was, however, clearly indicated, and in this connection a curious fact was stated by Dr. Snow, of England, who had devoted so much study to the use of anæsthetics, that as far back as thirty-five or forty years ago a case of tetanus was reported to have been cured by the inhalation of sulphuric ether. In reply to his friend, Professor Gibson, he would remark that the onus of proof must rest with those who denied the idiopathic origin of the affection. The lesion, however slight, must really be found to exist. If we are to admit the most inconspicuous peripheral abrasion as an all-sufficient cause of such speedily deadly consequences, it might well be asked why was tetanus so very rare an accident in any of its forms? why was it not met with constantly and everywhere? For his part, he saw no relation whatsoever between the extent of the injury and the vehemence and fatality of the results—no interdependence of cause and effect. Besides, the concurrent testimony of many writers shows that climate so far influences the development of this malady, that it is comparatively frequent, if not of endemic prevalence in certain latitudes. At least one form of it—*trismus nascentium*—is so common in India as to destroy one half the infants who are born in those regions. Here some idiopathic cause must obtain; for though it was customary to refer *lock-jaw* to injury of the cord, this he never admitted, for the cord had no nerves. The potency of climatic influence, in producing spontaneous development of spasms, was shown in the interesting fact stated by Dr. Kane in his Arctic Expedition, where he observed that all of his dogs and some of his men were seized with tetanoid spasms, of which they all died. Dr. M. said if he had referred puerperal tetanus to the *moulting* of the uterine surface, it was to show that so considerable a breach of surface, though a physiological act, might produce changes in the blood. Not only was the neck of the womb fissured, but there was always laceration of the posterior commissure of the vulva—the *fourchette*. There was practical utility in the inquiry into the probable causes and differences of these forms of the disease. How are we to apply curative means if we know nothing of the pathogeny of the affection? If it be dependant always upon injury to a nerve, we must amputate or cut around the nerve; if to change in the blood, iron, quinine, wine, etc., should be used, which latter treatment, of late, seemed to prove more successful.

Surgeon Gibson replied that nothing better exemplified the inconclusiveness of the argument in favor of the idiopathic nature of tetanus, based upon apparent absence of any lesion, than the ascertained history of certain cases of hydrophobia—a disease known to depend upon introduction into the blood of a virus derived from a rabid animal—in which cases hydrophobia had apparently arisen spontaneously. A woman died of what was called *idiopathic hydrophobia*—for it was positively ascertained that she had never been bitten by any dog, but it was afterwards discovered that she had used, in some way, a rope which had served to confine a rabid dog. Here it was evident the virus which can remain so long dormant in the system at times, must have lain latent in the coils of the rope, and must have come in contact with some abraded surface. Another woman had hydrophobia from the scratch of a cat's claw. Such instances showed how difficult it was sometimes to trace each link in the change of causation, and these examples strengthened his belief in the non-existence of idiopathic tetanus. He differed as to there being no nerves in the cord; the blood-vessels of the cord must be accompanied by nerves, for these are found everywhere along the coats of the vessels.

Surgeon Michel begged permission to reply to Prof. G.'s last remark. The umbilical cord and placenta, constructed to subserve a temporary purpose and disappear as relicts of fœtal structure, were, under such a plan, unprovided with those elements of organization possessed by permanent organs. There were not only no nerves, but also no lymphatics in either cord or placenta. The evolution of these parts in the embryo explained the reason of this exception. How were these parts formed?—the allantois was a provisional structure, only designed to convey vessels to the surface of the chorion, and through its villosities to the mucous membrane of the womb, thereby establishing an ovo-uterine connection. During this stage, the amnion folds around it, and the vitelline sac with its omphalo-mesenteric vessels, which structure soon entirely disappears, while the pedicle of the allantois contracting into the urachus retains only these embryonal vessels surrounded by the gelatin of Wharton. Therefore, as a needless expenditure of structure, no filaments of the sympathetic nerves nor sympathies were traceable along the vessels of the cord, nor their convoluted capillaries which form a placenta.

Surgeon Brewer saw, when a student, a case of tetanus which was supposed to be idiopathic, but on proceeding to make the *post-mortem* examination, it was discovered that the subject had worn a moxa for some time. Believing, that like insanity with regard to the brain, so in tetanus in relation to the spinal cord, there existed a predisposition or constitutional proclivity to the disease. The negro was more disposed to tetanus, perhaps, from a greater nervous susceptibility, together with being more exposed and less warmly and comfortably clad. He had seen spasms brought on by the use of liquors—whiskey spasms, as they were called. In treatment, we do little but assist nature and allow the disease to wear itself out. In cases reported cured by chloroform, it was doubtful whether the chloroform cured or only assisted nature.

Surgeon McCaw considered the term *idiopathic* tetanus one which should be banished from our nomenclature, as he did not see how the same disease could exist in two forms so different in their results, as to prove invariably fatal at one time, and almost always end in recovery at another. What was described as idiopathic tetanus, seldom terminated in death, while traumatic tetanus was always fatal. He thought two very different conditions existed in these affections described as identical. True tetanus was always of traumatic origin. But when we speak of idiopathic spasms, reference is made to a blood disease. The spasms arising from strychnine, are clearly due to the introduction of that poison into the circulation. In whiskey spasms, an excess of carbonic acid in the blood is produced by the introduction of *fusel oil* into that fluid. While, again, in the spasms described by Dr. Kane, the phenomenon was due to the retention of carbonic acid in the blood, owing to the absence of light. Under all such instances, therefore, the blood is at fault. Possibly, in so-called idiopathic tetanus, some change occurs in the blood, not from extremes of heat or cold, but from *sudden alternations* of temperature. In certain latitudes, such morbid manifestations were more frequent than in milder or more regular climates, and these influences of temperature were exerted in developing even true tetanus; hence, lock-jaw destroys more infants in one part of the world than in another. He believed this infantile disease to be essentially traumatic, depending upon the lesion about the navel, resulting from the sloughing of the cord, as it generally comes on about the ninth day.

*Cold-Water Dressings.* By Surgeon J. B. Read.

Surgeon Read read a report on cold-water dressings, embodying the statements and opinions of the many surgeons who had replied to inquiries on this subject.

The reporter referred to the three ways in which this agent was generally applied in hospital and private practice, by cloths or lint wet with cold water; by irrigation or *guttatim;* and by wetted cloths, covered with a greased rag or oiled silk to prevent evaporation, or still more elegantly by the application of wet spongio piline. This last method of using cold water, converts it gradually into a warm-water dressing. The effect contemplated by the use of this agent is reduction of temperature, and sedation. This result is obtained by the direct constringing power of cold, which renders latent the abnormal quantity of caloric incident upon the increased flow of blood in the part, and by the evaporation caused by this increased heat, and the constant currents of air. To produce this result, cold water must be continuously applied and for a length of time corresponding to the strength of the patient, and the local hyperœmia that obtains. The benefit of the treatment consists in not using it too soon or too long; but only in modifying and restraining the inflammatory process.

Cold, in constricting the capillaries, drives the blood from the surface, to which it immediately returns, if it be discontinued, with increased force, producing a glow both pleasant and healthful. The same effect occurs if cold water be intermittingly used, at intervals long enough to permit of its evaporating, and of the drying of the parts. By this method much inconvenience, and by its persistence even fatal injury, may ensue. The constant change from depression to excitement in the capillaries, produced by the reflux and flux of blood, ends in stagnation of the blood in the part, or may proceed to an extent in which the death of the tissue will ensue. A certain amount of inflammation must be present in order that the wound may progress favorably, and it is in modifying and measuring this action that cold water applications are especially useful, for repair of the parts may be prevented by too great, and delayed by too little, action. After receipt of a wound, it is more than probable that there is a stasis of the blood in the vessels for some little distance from it, which lasts for several days. This varies with the tissue, and says Paget: "This may be the brooding time either for good or evil." Now, this condition of the injured part is surely to be taken into consideration in the use of our applications to the wound. Nature, shocked by the injury, thus guards herself from more serious consequences, stays pressing and immediate danger of hemmorrhage by producing stagnation, and resting quietly, gathers her forces slowly and temperately, for the repair of the injury done; and much of the perfection of this effort depends on her being left quietly to work out the result in her own way. The point of practical importance is to distinguish accurately, when this stasis ceases and inflammation sets in, for this is the time for the continuous use of the agent. Men worn out by excessive excitement, badly fed and hardly overtasked by long marches and sleepless nights, dispirited or demoralized by defeat, cannot be in the same condition, as regards the healing of their wounds, as those who are well fed, who have suitable repose and are elated by the enthusiasm of victory. In the one case all depressing agents should be studiously avoided, until, at least, they are imperiously called for; in the other case, these would be of use at a much earlier period. This effect of the condition of the patient's system, was brought forcibly to mind after the seven days' battles around this city. In the hospital under our charge all wounds were treated with cold-water dressings, and such was the assiduity of the ladies, who at that time volunteered their aid so kindly, that the wounds were kept constantly drenched. The result was that the suppurative process was much delayed, and the necessary separation of the injured parts postponed to such a degree, that cold water as a constant application had to be discontinued; for from this indiscriminate drenching there was much greater destruction of tissue than we have since seen under a less vigorous treatment.

Wounds in some situations and of certain characters respond differently to a cold-water treatment. Wounds of the face, and those penetrating the cranial cavity, do well with cold-water dressings; while its use, in wounds penetrating the abdominal and thoracic cavities, is hardly compatable with sound judgment. The law that governs the lesser and greater fatality of amputations in the extremities, seems to obtain also in the use of cold water. It appears questionable, also, whether, in gun-shot wounds passing through large surfaces and great depths of muscles, cold water should be continuously applied. Its action, where the wound is superficial in its extent, is easily appreciated, but it cannot be equally efficient when applied to a deep wound, as it is separated from the greater part of the injured textures by intervening muscular masses, its repellent effect increasing congestion in the deeper portions of the track.

The demand for its use in wounds of joints is imperative; synovitis, the ingress of air into the sac; and all the train of evil consequences call for the advantageous use of cold-water *guttatim.* Cold and chillness always indicate the suspension of the agent. There are cases, however, irrespective of any such sensations, in which these dressings should be changed, and when the granulations become pale and flabby, assuming a greasy look, with a doughy feel of the surrounding tissues, the general practice has been to discontinue cold dressings after the establishment of suppuration, but should no unpleasant chillness or deleterious effects be experienced, these may, even throughout this stage, be continued with the effect of controlling the discharges and keeping the wound clean. This treatment, however, is perhaps inapplicable to extensive shell wounds, in which large lacerated surfaces are exposed. The depressing effects are such as to retard the sloughing process, which is often rendered troublesome and excessive.

The method of covering the wetted cloths with oilsilk or a greased rag is of general utility, as it places wounds in the condition most essential for their well doing, and fairly shields them from the injurious effects of the air. The parts are kept moist and soft, and the hardening of effused blood is prevented, relaxation of the capillaries is induced and we realize the advantages derived from a warm bath. We are of opinion, that, in the majority of cases of compound comminuted fractures, as also in penetrating wounds of the thorax and abdominal cavities, it is the only mode of application admissible. From the perusal of the documents before us, we have learned of no injurious effects from the cold-water treatment when properly used. Neither gangrene, (sloughing phagedæna), nor erysipelas has resulted. The transient vesicular or papular eruption produced by such dressings is too evanescent to be termed an injury. Scrupulous care should be paid to cleanliness, by each patient using his own basin and sponges while resorting to this mode of treatment. The cloths previously used should be burned where grangrene has prevailed to any extent.

We submit therefore:

That cold water is invaluable in the treatment of gun-shot wounds. That it may be used in three ways—by direct application, dripping, and by applications covered so as to prevent evaporation and exclude air. That each of these methods has its especial good, according to the condition of the wound and the part of the body injured. That the first methods are not as generally applicable as the third, and should only be used in specific cases to produce certain effects, and that as soon as these effects are produced, they should be discontinued. The third method is of most general utility, and in order that the proper result may not be interfered with, the application should not be made oftener than three or four times in twenty-four hours.

